# Supplementary material for: A unified model of short- and long-term plasticity: Effects on network connectivity and information capacity
Source: PLoS Comput Biol. 2026 Sep 2;22(9):e1014730. doi: 10.1371/journal.pcbi.1014730 (PMC13581215; doi:10.1371/journal.pcbi.1014730)
Supplement: Supplementary file 1 [file pcbi.1014730.s001.pdf]

S1: Supplementary Triplet model results

Iiro Ahokainen and Marja-Leena Linne, Tampere University

iiro.ahokainen@tuni.fi

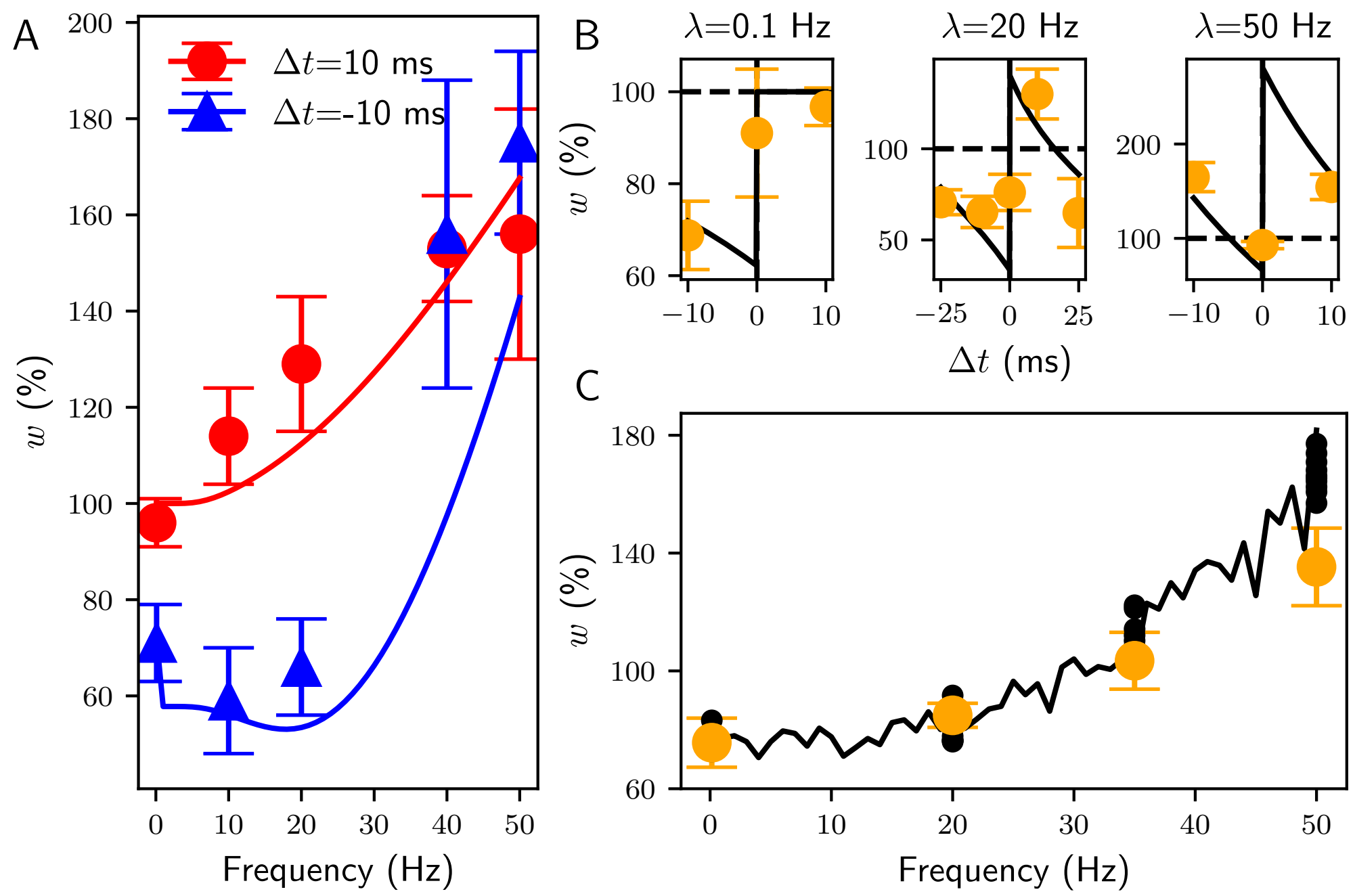

Fig A. Minimal all-to-all triplet model [1] fitted to the long-term plasticity induction protocol [2]. As in [1], $\tau_c = 16.8$ ms and $\tau_- = 33.7$ ms were fixed, and the remaining parameters were fitted. The fitted parameters and the fitting error E are: $A^+ = 0.0285$, $A^- = 0.0076$, $\tau_+ = 35.09$ ms, and $E = 914.8$. (**A**) Change in w in response to varying interspike intervals, resulting in a nonlinear frequency response with fixed $\Delta t = \pm 10$ ms. Dots and triangles, with standard deviations, represent data from [2], and the lines indicate the fits. (**B**) The update window with varying Δt at three different frequencies (0.1 Hz, 20 Hz, and 50 Hz), shown from left to right. (**C**) Model fit to the randomized Δt. As in [2], Δt was drawn from a Gaussian distribution with a mean of zero and a standard deviation of 7 ms. Orange error dots show the experimental data, and black dots show all the realizations of model fits. The black line shows one example fit across the entire frequency range.

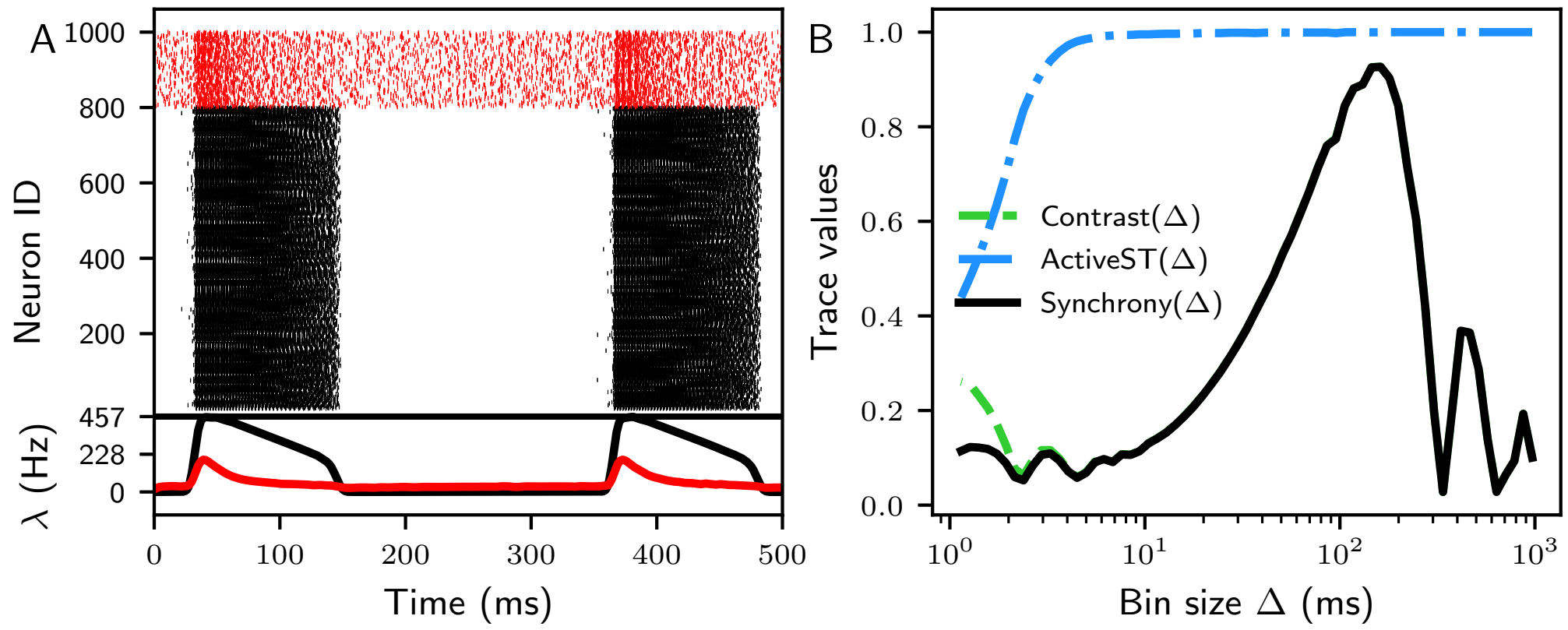

Fig B. Activity of the uncoupled TM-Triplet network after 30 minutes of self-organization, exhibiting network bursting and synchronization. (**A**) Raster plot of the uncoupled TM-Triplet network after 30 minutes of self-organization. Black markers indicate spike times of excitatory neurons, and red markers indicate spike times of inhibitory neurons. At the bottom, the averaged population activity λ is shown for excitatory neurons (black) and inhibitory neurons (red). Due to the runaway potentiation of E $\rightarrow$ E connections, the uncoupled TM-Triplet network exhibits superficially high firing rates for long times during bursts. Both the excitatory and inhibitory populations show bursting activity. (**B**) Spike-contrast metric [3] for the excitatory spikes shown in (**A**). Synchrony is measured as a product of Contrast and ActiveST (active spike trains). The maximum synchrony of 0.93 is achieved with a bin size of $\Delta = 145.4$ ms.

References

1. Pfister JP, Gerstner W. Triplets of Spikes in a Model of Spike Timing-Dependent Plasticity. The Journal of Neuroscience. 2006;26(38):9673–9682. doi:10.1523/jneurosci.1425-06.2006.

2. Sjöström PJ, Turrigiano GG, Nelson SB. Rate, Timing, and Cooperativity Jointly Determine Cortical Synaptic Plasticity. Neuron. 2001;32(6):1149–1164. doi:10.1016/s0896-6273(01)00542-6.

3. Ciba M, Isomura T, Jimbo Y, Bahmer A, Thielemann C. Spike-contrast: A novel time scale independent and multivariate measure of spike train synchrony. Journal of Neuroscience Methods. 2018;293:136–143. doi:10.1016/j.jneumeth.2017.09.008.
