# Supplementary material for: A unified model of short- and long-term plasticity: Effects on network connectivity and information capacity
Source: PLoS Comput Biol. 2026 Sep 2;22(9):e1014730. doi: 10.1371/journal.pcbi.1014730 (PMC13581215; doi:10.1371/journal.pcbi.1014730)
Supplement: Supplementary file 3 [file pcbi.1014730.s003.pdf]

S3: Analysis of inhibitory activity

Iiro Ahokainen and Marja-Leena Linne, Tampere University

iiro.ahokainen@tuni.fi

This supplementary section presents additional analyses of inhibitory network activity for both the SL-STDP and uncoupled TM-Triplet models under different self-organization conditions. We characterize inhibitory dynamics using firing rate statistics and coefficient-of-variation (CV) distributions. The results highlight differences in inhibitory firing regularity and self-organization between the two plasticity models, particularly in the presence of excitatory-to-inhibitory plasticity.

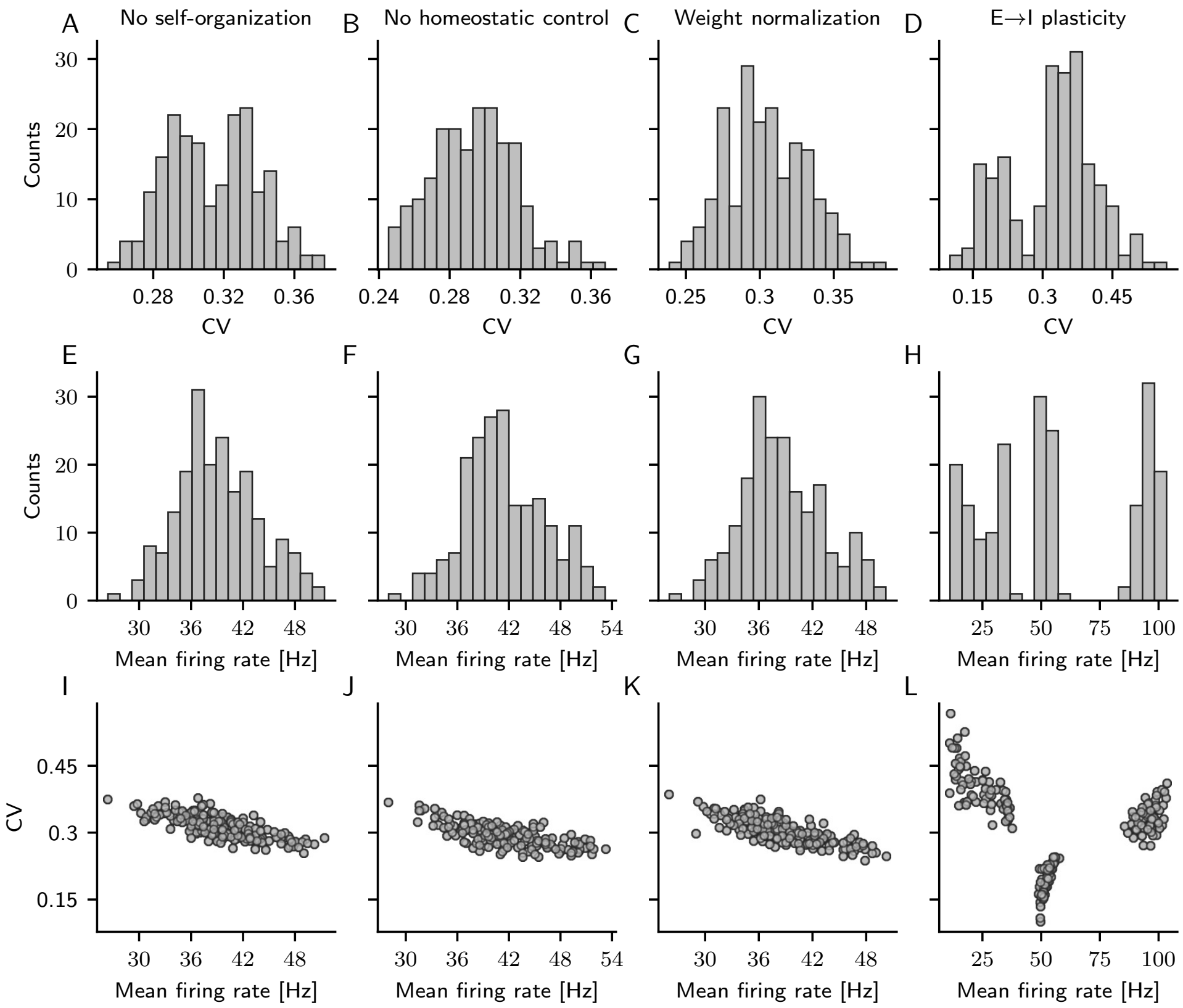

Fig A. SL-STDP networks' inhibitory activity statistics with different conditions. The top row (**A-D**) shows Coefficient of Variation (CV) histograms for all inhibitory neurons evaluated over five seconds of network activity. The middle row (**E-H**) shows the mean firing rates for all inhibitory neurons. The bottom row (**I-L**) shows the CV values as a function of the mean firing rates. The first column shows the network statistics prior to self-organization, meaning that the same statistics also apply to the uncoupled TM-Triplet model before self-organization. The mean firing rates of inhibitory neurons remain fairly similar when no homeostatic control or weight normalization is applied (**E-G**). (**H**) In contrast, E $\rightarrow$ I plasticity clusters the firing rates mimicking excitatory neurons' activity (see Fig 9 in the main article), and the CV histogram shows two peaks (**D**). (**L**) Each cluster has preferred range of CV values. Interestingly, the lowest CV range is with the cluster around 50 Hz. Note that this coincides with the network oscillation frequency.

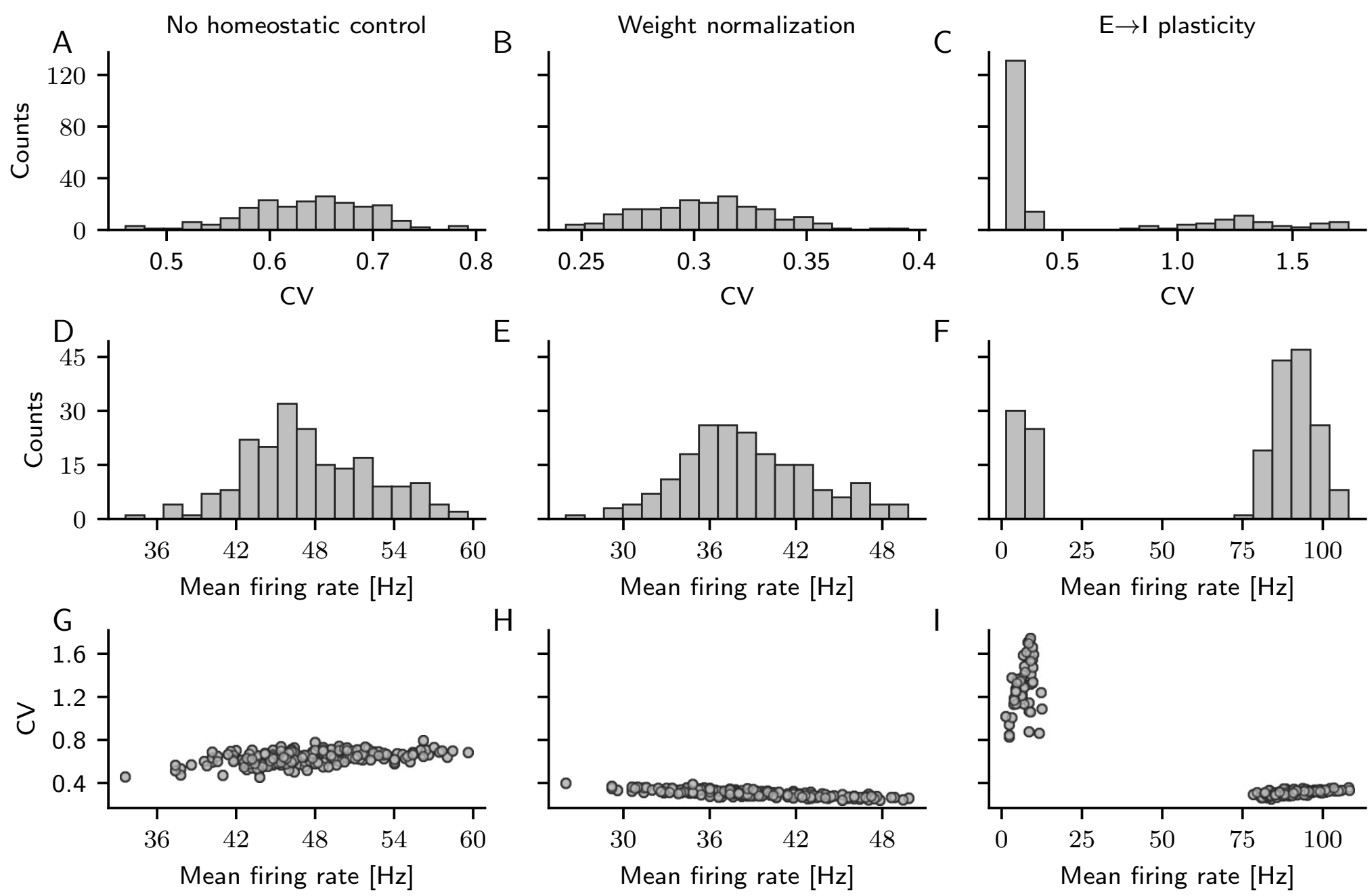

Fig B. Uncoupled TM-Triplet networks' inhibitory activity statistics with different conditions. The top row (**A-C**) shows Coefficient of Variation (CV) histograms for all inhibitory neurons evaluated over five seconds of network activity. The middle row (**D-F**) shows the mean firing rates for all inhibitory neuron. The bottom row (**G-I**) shows the CV values as a function of the mean firing rates. CV values for the case without homeostatic control (**A**) are elevated compared to the SL-STDP network (Fig AB). (**F**) E $\rightarrow$ I plasticity clusters inhibitory firing rates into two clusters following the excitatory neurons' activity (see Fig 9 in the main article). (**C**) Small amount of CV values reach over one, indicating irregularity in the firing.
