# Supplementary material for: A unified model of short- and long-term plasticity: Effects on network connectivity and information capacity
Source: PLoS Comput Biol. 2026 Sep 2;22(9):e1014730. doi: 10.1371/journal.pcbi.1014730 (PMC13581215; doi:10.1371/journal.pcbi.1014730)
Supplement: Supplementary file 4 [file pcbi.1014730.s004.pdf]

S4: Supplementary information capacity results

Iiro Ahokainen and Marja-Leena Linne, Tampere University

iiro.ahokainen@tuni.fi

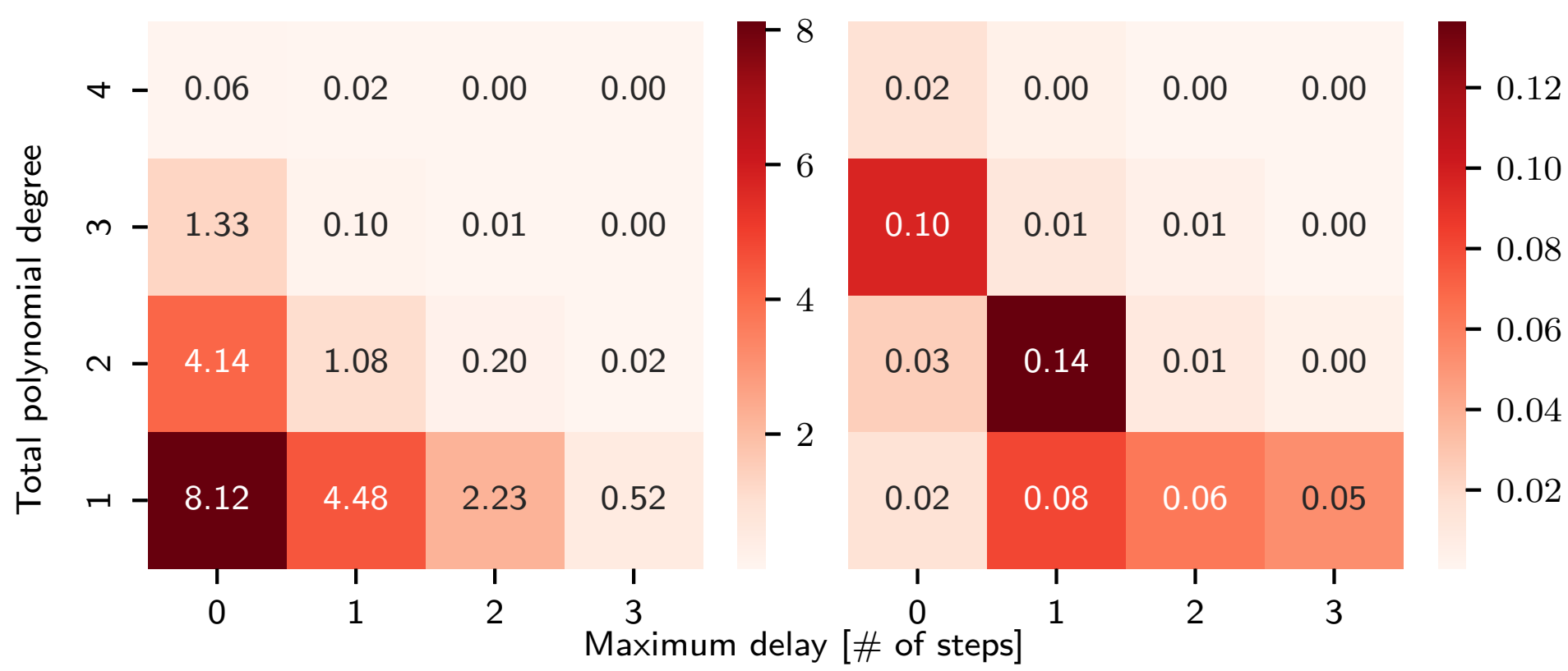

Fig A. Absolute test capacity values for the baseline network. (**Left**) Mean capacity values over 10 different random initializations of the network. A capacity value of 10 is the theoretical maximum. Increasing total polynomial degree and maximum delay correspond to more difficult working memory tasks. (**Right**) Corresponding standard deviations for each cell shown on the left.

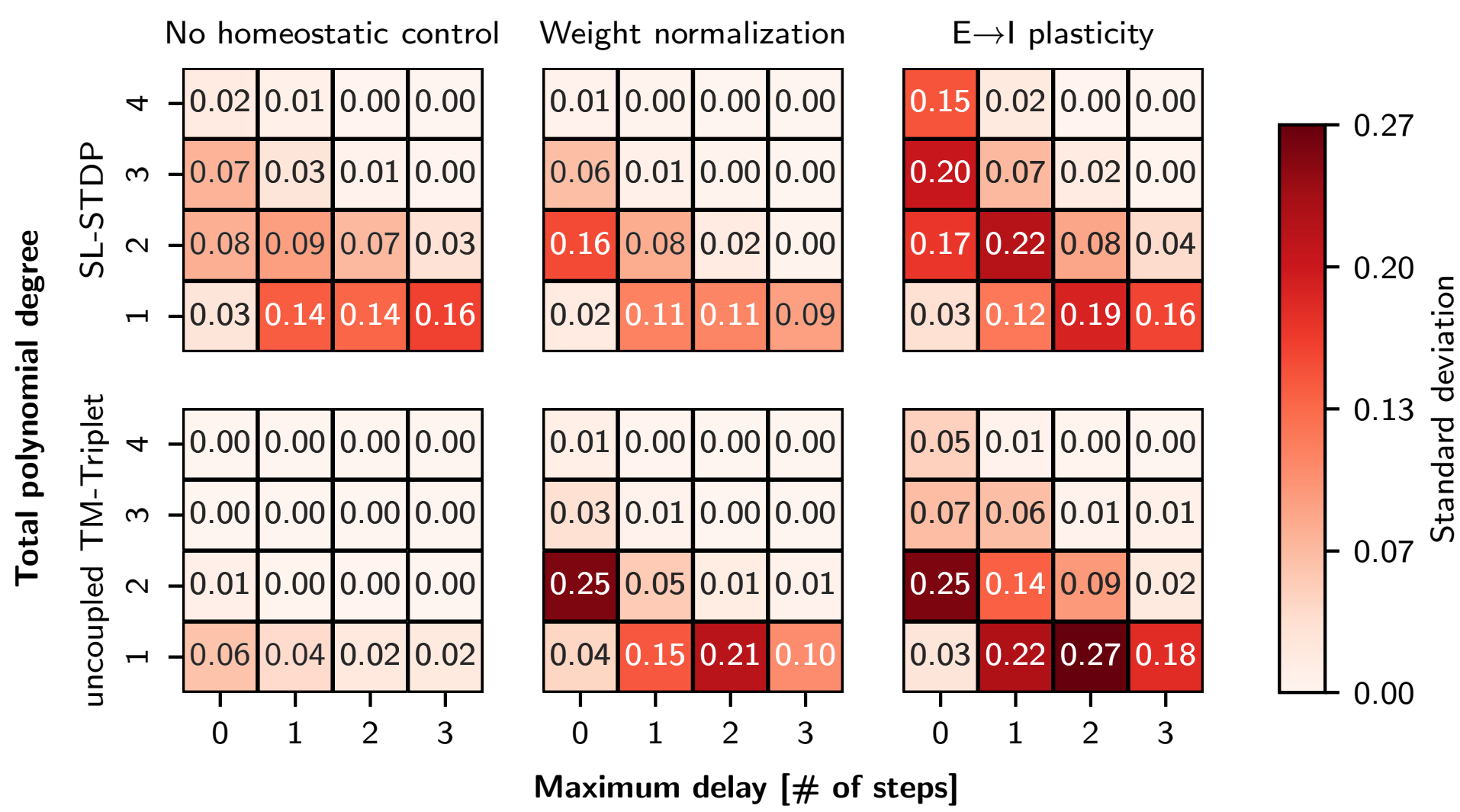

Fig B. Standard deviations of test capacities with different homeostatic mechanisms. The standard deviations of the absolute capacity values over 10 runs are shown for each combination of plasticity model and homeostatic control mechanism.

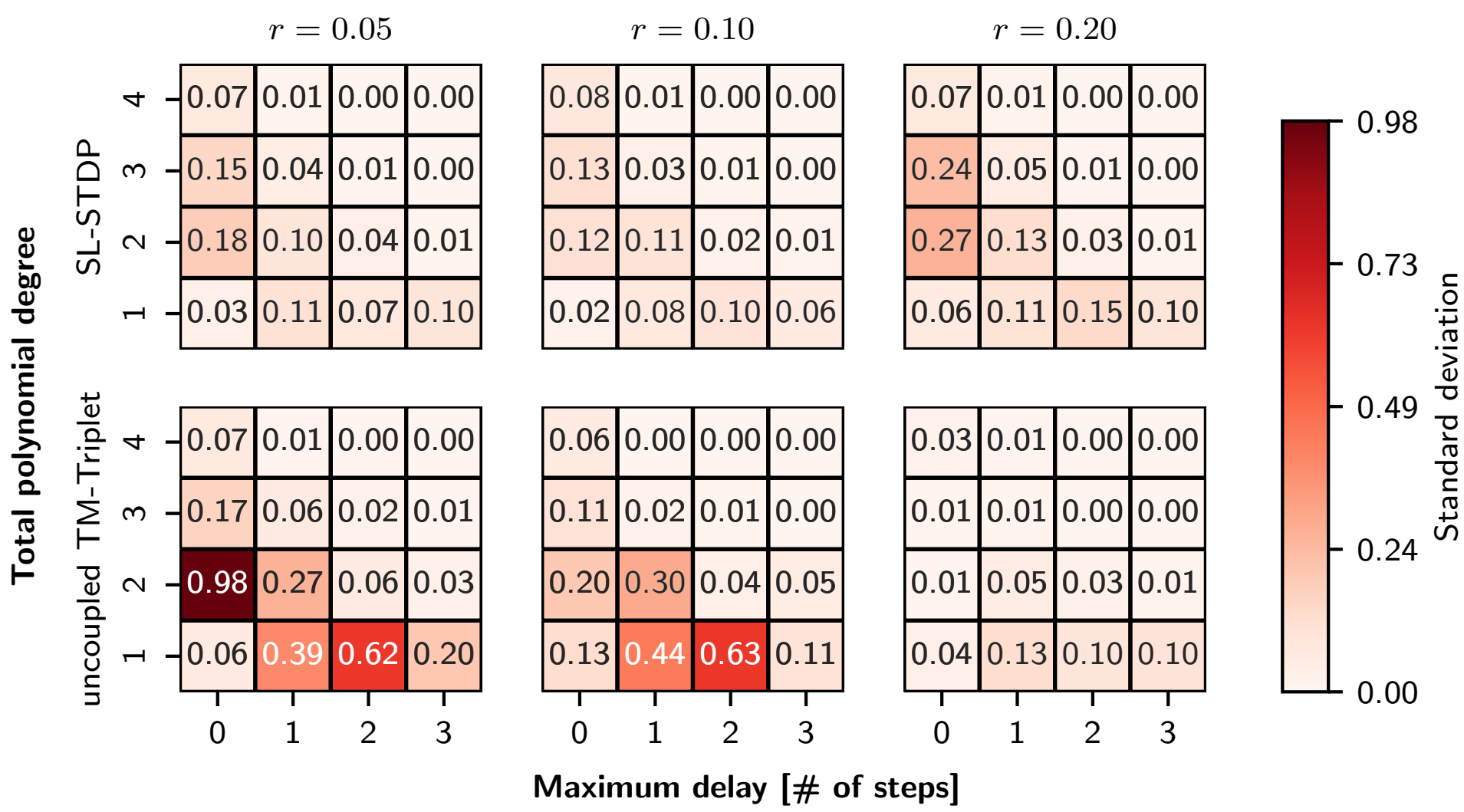

Fig C. Standard deviations of test capacities with different ratios of facilitating and depressing synapses. The standard deviations of the absolute capacity values over 10 runs are shown for each combination of plasticity model and ratio r between facilitating and depressing $\mathrm{E} \rightarrow \mathrm{I}$ synapses. Overall, the SL-STDP model shows less deviation than the uncoupled TM-Triplet model in almost all cells.
