# Supplementary material for: A unified model of short- and long-term plasticity: Effects on network connectivity and information capacity
Source: PLoS Comput Biol. 2026 Sep 2;22(9):e1014730. doi: 10.1371/journal.pcbi.1014730 (PMC13581215; doi:10.1371/journal.pcbi.1014730)
Supplement: Supplementary file 6 [file pcbi.1014730.s006.pdf]

S6: Synapse models' parameter comparison and network simulation parameters

Iiro Ahokainen and Marja-Leena Linne, Tampere University

iiro.ahokainen@tuni.fi

Table A. Comparison of depressing synapse parameter values for the SL-STDP model and the uncoupled TM-Triplet model network simulations. These parameters are used in all E $\to$ E connections and also depressing E $\to$ I connections. In simulations where E $\to$ I connections are not plastic, then $f = 0$.

Parameter	Description	SL-STDP	Uncoupled TM-Triplet	Units
τ_r	Depression time constant	200	200	ms
τ_f	Facilitation time constant	50	50	ms
U	Release probability increment per presynaptic spike	0.2029	0.2029	–
U_b	Baseline release probability	0.7019	0.7019	–
τ_y	Time constant of presynaptic spike efficacy $y(t)$	16.54	16.54	ms
τ_c	Time constant of Triplet presynaptic trace $c(t)$	–	16.8	ms
τ_-	Time constant of LTD postsynaptic trace $z_1(t)$	100.0	33.7	ms
τ_+	Time constant of LTP postsynaptic trace $z_2(t)$	63.30	35.09	ms
Z_1	Gain control of LTD postsynaptic trace	0.4102	–	–
Z_2	Gain control of LTP postsynaptic trace	0.1158	–	–
A_-	LTD update amplitude	0.0166	0.0076	–
A_+	LTP update amplitude	0.8499	0.0285	–
f	Learning rate	10.96	10.96	pA

Table B. Comparison of facilitating synapse parameter values for the SL-STDP model and the uncoupled TM-Triplet model network simulations. These parameters are used in facilitating E $\to$ I connections.

Parameter	Description	SL-STDP	Uncoupled TM-Triplet	Units
τ_r	Depression time constant	50	50	ms
τ_f	Facilitation time constant	500	500	ms
U	Release probability increment per presynaptic spike	0.15	0.15	–
U_b	Baseline release probability	0.15	0.15	–
τ_y	Time constant of presynaptic spike efficacy $y(t)$	16.54	16.54	ms
τ_c	Time constant of Triplet presynaptic trace $c(t)$	–	16.8	ms
τ_-	Time constant of LTD postsynaptic trace $z_1(t)$	100.0	33.7	ms
τ_+	Time constant of LTP postsynaptic trace $z_2(t)$	63.30	35.09	ms
Z_1	Gain control of LTD postsynaptic trace	0.4102	–	–
Z_2	Gain control of LTP postsynaptic trace	0.1158	–	–
A_-	LTD update amplitude	0.0166	0.0076	–
A_+	LTP update amplitude	0.8499	0.0285	–
f	Learning rate	10.96	10.96	pA

Table C. Network simulation specific parameters.

Parameter	Description	Value	Units
w_{init}	Initial postsynaptic strength	$10.96 \pm 1.096^{\dagger}$	pA
w_{max}	Maximal postsynaptic strength	109.6	pA
t_{delay}	Spike time delay	[0.1, 1.0[*	ms
p	Initial connection probability	0.3	-
λ_{θ}	Threshold Poisson noise rate	8968	Hz
g	Initial inhibitory synaptic strength ratio to excitatory synaptic strength	5	-
N^{exc}	Number of excitatory neurons	800	-
N^{inh}	Number of inhibitory neurons	200	-

† Sampled from Gaussian distribution mean±std.
* Sampled from uniform distribution.
